# Introduction: *Drosophila*—A Model System for Developmental Biology

**DOI:** 10.3390/jdb5030009

**Published:** 2017-09-20

**Authors:** Nicholas S. Tolwinski

**Affiliations:** 1 Division of Science, Yale-NUS College, Singapore 138527, Singapore; Nicholas.Towlinski@yale-nus.edu.sg; Tel.: +65-6601-3092; 2 Department of Biological Science, National University of Singapore, Singapore 138615, Singapore

**Keywords:** *Drosophila*, model organisms, development, genetics

*Drosophila melanogaster*, known colloquially as the fruit fly, remains one of the most commonly used model organisms for biomedical science. For more than one hundred years, the low cost, rapid generation time, and excellent genetic tools have made the fly indispensable for basic research. The addition of numerous molecular tools has allowed the model system to keep up with the latest advances. In this issue, various authors provide examples of how *Drosophila* is currently being used, and what directions they think the system is moving in. From human disease modeling to the dissection of cellular morphogenesis and to behavior and aging, this issue examines the current uses of flies, and the influence of fly research on other models.

Why the fly was chosen for research may prove difficult to pin down historically, but its rise to prominence is well documented [1]. Thomas Hunt Morgan used the fly to prove the chromosomal theory of inheritance showing that the *white* gene resided on the X chromosome, a finding for which he received a thoroughly deserved Nobel Prize [2]. He and his protégés then went on to define many of the principles of genetics, including the effects of X-rays on mutation rates, for which Hermann Muller also won the Nobel Prize [3]. From these discoveries came the generation of balancer chromosomes, a set of specialized chromosomes that prevent recombination through a series of DNA inversions. These tools allow researchers to maintain complex stocks with multiple mutations on single chromosomes over generations, an advance that made flies the premier genetic system [4]. Genetic tools such as these led to ever more complex genetics and more complex problems being addressed. For example, Seymour Benzer, famous for working out the topology of genes using bacteriophage, turned to *Drosophila* to study the influence of genes on behavior [5]. His work greatly contributed to one of the great debates in biology, namely how much do genes contribute to higher brain function, an advance he accomplished using simple genetic and complex mosaic experiments coupled with clever assays to observe interesting changes in behavior. 

The modern era of *Drosophila* research really took off when the embryo was analyzed in depth for genes involved in its development [6]. This work launched many fields of developmental biology and led to another *Drosophila* Nobel Prize [7]. The basic discovery was that discrete genes regulated different aspects of development. Many of these genes turned out to be homologous to those involved in human development and disease. These genes had been conserved over millions of years of evolution and could be studied easily and rapidly in flies. This led to a boom in the field as more and more researchers saw the potential of flies for asking basic and applied questions, and to the development of ever cleverer molecular tools to address these questions. For example, chemical mutagenesis was used for many years to generate new mutations that were screened for interesting phenotypes, followed by careful genetic mapping, a chromosome walk, and finally gene cloning [8]. Currently, the MiMIC transposon system is being applied to target all genes in the *Drosophila* genome, providing null mutations and a platform to land protein tagging, gene expression tracking, and many other functions through an exon swapping approach [9]. These, in conjunction with CRISPR/Cas9 knockout/knockin and overexpression strategies [10], allow the inactivation, tagging, and overexpression of any gene in the genome within weeks of starting a project. Using this approach, any gene or even allele related to human disease can be studied in flies. In fact, these approaches, and many others, have been put together into a genetic toolkit to test human disease genes in *Drosophila* [11]. 

As research budgets shrink in real terms, it is easy to overlook basic research in such an abstract and annoying animal as the fruit fly. Model organism research can be an easy target for a quick joke by a politician or journalist, and it is much easier to justify research spending on humans or human-derived materials, as “translation” is much more obvious in such studies. However, human studies are enormously expensive and very slow, leaving model organism research as the best, cheapest way to study anything more complex. In this issue, the authors will explore recent developments in fly research and compare them to the recent advances in other model organisms. This field remains vibrant and exciting, with labs using flies in drug discovery, bioengineering, regenerative biology, and medicine. The future for model organism research is bright.
